# The pathogenesis of diabetic kidney disease and the therapeutic potential of bioactive substances

**DOI:** 10.3389/fphar.2025.1669424

**Published:** 2025-11-24

**Authors:** Yan Chen, He Li, Qiuju Dai, Zhen Tan, Huihui Wu, Zhiyi Xu, Guangwei Wang, Yang Fang, Jie Luo, Chenghao Yu, Mingliang Huang, Cheng Peng

**Affiliations:** 1 School of Public Health, Chengdu University of Traditional Chinese Medicine, Chengdu, Sichuan, China; 2 State Key Laboratory of Southwestern Chinese Medicine Resources, Chengdu, China; 3 Sichuan Huashen Group Co., Ltd, Chengdu, China; 4 Chengdu Huasun Technology Group Inc, LTD, Chengdu, China

**Keywords:** diabetic kidney disease, pathogenesis, bioactive substance, mechanism, target point

## Abstract

Diabetic kidney disease (DKD), one of the common complications of diabetes, is a major contributor to chronic kidney disease (CKD) worldwide. Due to its high incidence and disability rates, DKD poses a serious threat to human health and represents a significant public health burden. Although current treatments, such as angiotensin-converting enzyme (ACE) inhibitors, are available, they remain limited in pharmacological effects. Medicinal plants are valuable resources for drug development, and their bioactive compounds have attracted considerable attention for their therapeutic potential in DKD. In this review, we systematically searched major scientific databases (e.g., PubMed) for studies published within key timeframes relevant to each topic. Keywords such as “DKD and oxidative stress” and “diabetic nephropathy and bioactive substances” were used to identify high-quality original research and review articles closely aligned with the theme of this work. Based on the selected literature, we summarize the pathogenic mechanisms of DKD and elucidate the therapeutic effects and mechanisms of bioactive substances—including polyphenols, peptides, polysaccharides, and flavonoids—in its treatment. A comparative analysis is also presented to provide a foundation for future pharmacological research on DKD.

## 1 Introduction

Diabetic kidney disease (DKD) is a chronic kidney disorder that has become one of the most devastating complications of diabetes worldwide and a leading cause of end-stage renal failure. DKD accounts for 30%–50% of all chronic kidney disease (CKD) cases, affecting 285 million patients worldwide (Webster et al., 2017). Over one-third of individuals with type 1 diabetes and nearly half of those with type 2 diabetes are affected by this condition (Młynarska et al., 2024). The pathogenesis of this disease involves cellular autophagy, inflammatory responses, and oxidative stress. Pathological manifestations include thickening of the glomerular basement membrane, progressive loss of podocytes, and dilatation of the renal interstitial matrix. As the disease progresses, glomerulosclerosis and tubulointerstitial fibrosis develop, leading to a gradual decline in renal function and ultimately resulting in end-stage renal disease (ESRD).

As the prevalence of diabetes increases, so does the prevalence of DKD. DKD is strongly associated with all-cause mortality and cardiovascular mortality, significantly increasing mortality rates among patients with DKD (Di Lullo et al., 2015; Afkarian et al., 2013). Although treatments such as angiotensin-converting enzyme (ACE) inhibitors and angiotensin II receptor blockers (ARBs) are currently available, limitations still exist (Barrera-Chimal et al., 2022). Therefore, there is an urgent need to elucidate the molecular basis of DKD development and progression, and to develop novel therapeutic strategies and innovative drugs.

Medicinal plants serve as readily available resources for drug discovery. In recent years, mounting evidence has demonstrated the therapeutic potential of bioactive compounds for DKD, attracting widespread public attention. Curcumin extracted from *Curcuma L.* has demonstrated protective effects against DKD due to its potent anti-inflammatory and antioxidant properties, leading to the development of a range of established dietary supplement products such as Doctor’s Best Curcumin Phytosome. Resveratrol exerts a positive effect in treating DKD by lowering blood glucose levels through multiple complex pathways (Ke-Xue et al., 2021). Related products such as Healthy Care Resveratrol are already available on the market.

For this review, pertinent literature was identified through searches conducted on PubMed, Web of Science, and CNKI utilizing keywords such as “DKD” “oxidative stress” “bioactive substances” “autophagy” and “inflammation”. This paper thoroughly examines the mechanisms of action of bioactive compounds such as polyphenols, peptides, polysaccharides, and flavonoids in the treatment of DKD. It conducts comparative analyses of various aspects of these bioactive substances and summarizes their limitations, thereby providing a theoretical foundation and scientific basis for drug development and clinical treatment strategies targeting DKD.

## 2 Pathogenesis of DKD

### 2.1 Oxidative stress

Xiao-rong Wang et al. used a bibliometric study to find 4,076 publications in 755 journals on Oxidative stress (OS) and DKD-related literature during 2014–2024, with an increasing trend each year (Wang et al., 2024). This indicates a substantial growth in global interest regarding the influence of OS on DKD and stresses the essential need to investigate the pathogenic mechanisms of DKD from the molecular viewpoint of OS.

Leiming Sun found in a previous study that the activated JAK/STAT pathway may further stimulate the overproliferation and growth of glomerular mesangial cells (GMCs), leading to diabetic kidney injury. After the administration of isoglycyrrhizin, IL-6 and ICAM inflammatory cytokines were significantly downregulated, and the expression of p-STAT3 and p-JAK2 was significantly upregulated in DN kidney tissues (*p* < 0.05), accompanied by a nephroprotective effect, which confirms that isoglycyrrhizin ISO can protect the kidneys of rats in the acute DKD model by down-regulating the signaling pathway of JAK2/STAT3 (Sun et al., 2021).

During the development of DKD, OS activates a series of key signaling pathways that are intertwined and interact with each other to drive disease progression. The polyol pathway (Darenskaya et al., 2023) is stimulated by hyperglycemia with a significant increase in aldose reductase activity, which results in a massive conversion of glucose to sorbitol (Radenkovic et al., 2023). Frank R.N., Greene D.A., et al. have demonstrated through various studies that while the role of sorbitol in the polyol pathway is still debated, it plays a significant part in triggering diabetes (Singh et al., 2021).

The buildup of sorbitol in the cells leads to an increase in intracellular osmotic pressure, causing cellular osmotic damage. At the same time, activation of the polyol pathway also depletes large amounts of nicotinamide adenine dinucleotide phosphate (NADPH) and GSH synthesis, which further weakens cellular antioxidant capacity and exacerbates the extent of OS (Ma et al., 2023). In turn, OS further activates the polyol pathway, increasing sorbitol in the body and preventing it from being metabolized properly, creating a vicious cycle (Singh et al., 2021).

The protein kinase C (PKC) pathway is also activated in response to OS. Hyperglycemic state increases the production of di-lipoylglycerol (DAG) (Hayashi and Shirai, 2022), which is an endogenous activator of PKC. When PKC is activated, it activates a variety of downstream target proteins, such as NADPH oxidase, through a series of phosphorylation reactions (Li et al., 2024). Activation of NADPH oxidase leads to a further increase in ROS production (Singh et al., 2022), which exacerbates OS damage to renal cells. In addition, PKC regulates the expression of a variety of cytokines and growth factors, such as vascular endothelial growth factor (VEGF) and transforming growth factor-β (TGF-β) (Tanase et al., 2022), which are involved in pathological processes such as dysfunction of glomerular endothelial cells and proliferation of thylakoid cells, and increased synthesis of extracellular matrix during the development of DKD. The mitogen-activated protein kinase (MAPK) signaling pathway is another crucial pathway for OS activation (Zhang et al., 2020). The MAPK family includes several members, including extracellular signal-regulated kinase (ERK) and p38MAPK. Upon stimulation by OS, these MAPK members are activated and, through a phosphorylation cascade reaction, transmit signals into the nucleus to regulate the expression of relevant genes. For example, activated p38MAPK promotes the expression of inflammatory factors and apoptosis-related genes, leading to increased inflammatory response and apoptosis in kidney cells. ERK activation, on the other hand, may be associated with increased proliferation and extracellular matrix synthesis in tethered cells. The nuclear factor-κB (NF-κB) signaling pathway plays a key role in OS-induced inflammatory responses (Al-Malki et al., 2013). Normally, NF-κB binds to its inhibitory protein IκB (Chen et al., 2025) and exists in the cytoplasm in an inactive form. When cells are stimulated, such as by OS, IκB is phosphorylated and degraded, resulting in the release of NF-κB. Once NF-κB is inside the nucleus, it attaches to the promoter regions of specific genes, initiating the transcription and expression of inflammatory factors like TNF-α and IL-6, which leads to an inflammatory response. The inflammatory response can further damage kidney cells and promote the development of DKD (Yang et al., 2024).

**FIGURE 1:**
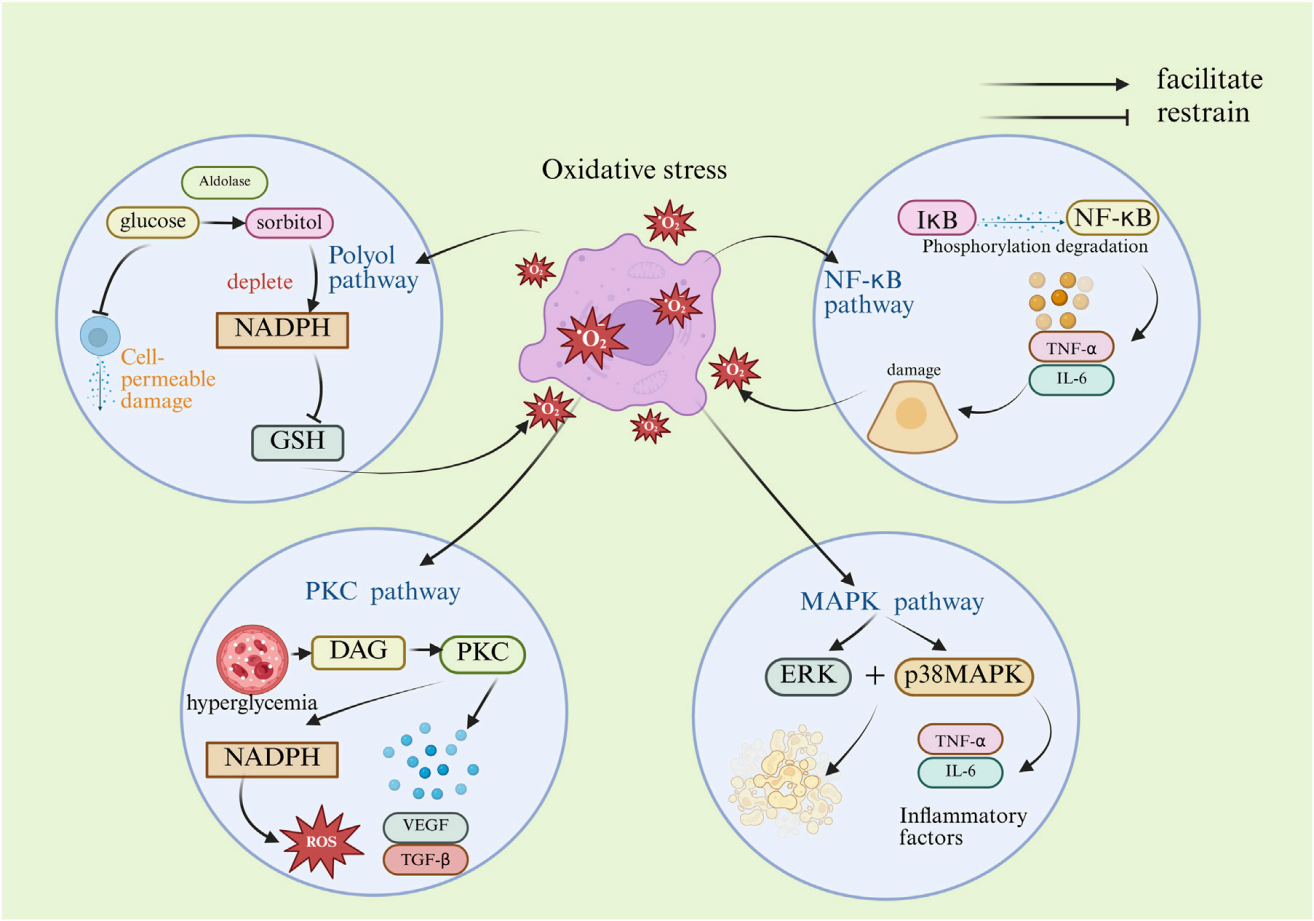
Mechanism diagram of oxidative stress-induced DKD. (DAG, diacylglycerol; ERK, extracellular regulated protein kinases; GSH, glutathione; IL-6, interleukin- 6; IκB, inhibitory proteins of nuclear factor kappa-B; NADPH, nicotinamide adenine dinucleotide phosphate; NF-κB, nuclear factor kappa-B; p38MAPK, p38 mitogen-activated protein kinase; PKC, protein kinase C; ROS, reactive oxygen species; TGF-β, transforming growth factor-β; TNF-α, tumor necrosis factor-α; VEGF, vascular endothelial growth factor).

In summary, oxidative stress activates a series of key signaling pathways that are intertwined and mutually influence each other, collectively driving the progression of DKD. Activation of the polyol pathway leads to cellular permeability damage and depletes the antioxidant NADPH, thereby exacerbating oxidative stress. Activation of the PKC pathway increases reactive oxygen species (ROS) production while promoting the expression of fibrotic factors, leading to glomerular dysfunction and fibrosis. Upon activation, p38MAPK and ERK in the MAPK pathway mediate inflammatory responses and apoptosis, as well as mesangial proliferation and fibrosis, respectively. The NF-κB pathway, as a core inflammatory pathway, activates the expression of numerous inflammatory factors upon its activation, directly causing damage to renal cells. Most of the current studies are on the effects of individual pathway mechanisms on OS, and whether these pathways have a combined effect on OS or whether they contribute specifically in different renal cell types has not been fully elucidated. Future studies could utilize cell-specific knockout animal models to precisely resolve the effects of different cellular sources of ROS on mechanisms and specific roles in DKD. The mechanism of oxidative stress in the pathogenesis of DKD is shown in Figure 1.

### 2.2 Abnormalities of glycolipid metabolism

Disorders of glucose and lipid metabolism can lead to insulin resistance. The liver is the main organ that regulates the balance of glucose and lipid metabolism and insulin resistance. Shuping Ruan et al. demonstrated in a clinical trial that people with T2DM and NAFLD have a 2.01 times higher risk of cardiovascular disease (Ruan et al., 2024). Impairment of insulin signaling in the liver, skeletal muscle and adipose tissue can lead to hyperglycemia, persistent high blood sugar levels can lead to cellular damage in the kidneys, potentially resulting in chronic kidney injury and speeding up the progression of DKD (Bai et al., 2025).

Previous studies have found that both endothelin-1 (ET-1) and adrenomedullin (ADM) affect glucose metabolism, and in a subsequent in-depth Mendelian randomization analysis, Chaterina Sujana et al. identified a SNP specific for CT-proET-1 in the EDN-1 gene (rs5370) and a SNP specific for MR-proADM in the ADM gene (rs2957692). rs2957692), which showed a positive correlation between CT-proET-1 and the incidence of type 2 diabetes (Sujana et al., 2022). Intracellular lipid metabolism and homeostasis are regulated by SREBPs, of which SREBP1 is a gene that controls lipid synthesis, and SREBP2 is a gene that controls cholesterol-related metabolism. In addition, when the storage and catabolic capacity of adipose tissue is exceeded due to excess lipids, lipids can be deposited in non-adipose tissues such as the liver and kidneys, causing lipotoxicity in these target organs, leading to insulin resistance, generation of reactive oxygen species (ROS), and endoplasmic reticulum stress, resulting in cellular damage and even death, which could result in the onset of DKD (Bai et al., 2025).

**FIGURE 2:**
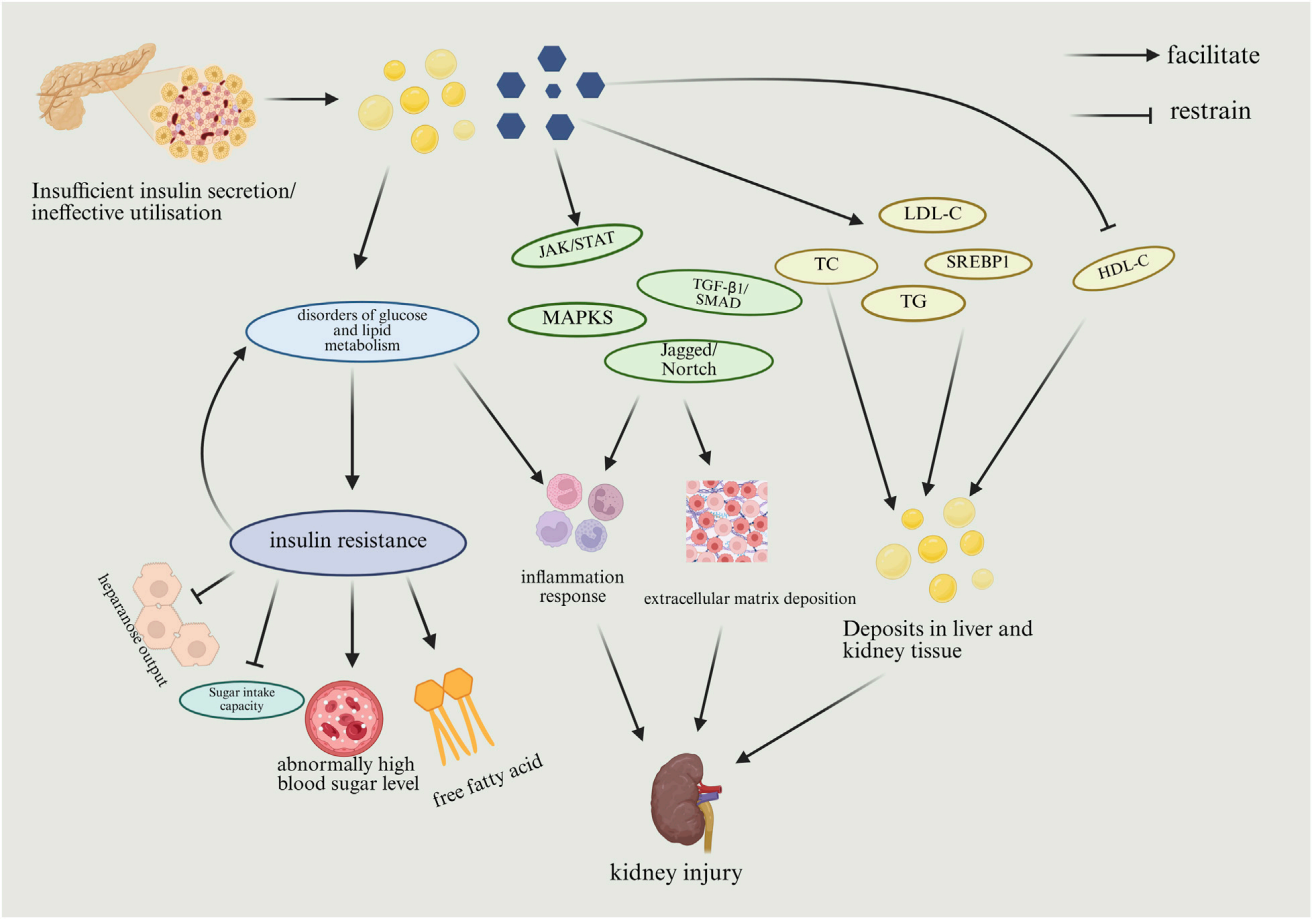
Mechanism of DKD due to abnormal glucose-lipid metabolism. (HDL-C, high density lipoprotein cholesterol; JAK, janus kinase; LDL-C, low-density lipoprotein cholesterol; MAPK, mitogen-activated protein kinases; SMAD, sma and mothers against decapentaplegic; SREBP1, sterol-regulatory element binding protein1; STAT, signal transducers and activators of transcription; TC, total cholesterol; TG, triglyceride; TGF-β1, transforming growth factor-β1).

Insufficient insulin secretion or failure to utilize insulin effectively leads to glucose and lipid accumulation. Lipid accumulation can lead to disorders of glucolipid metabolism, which can promote inflammation and insulin resistance. Insulin resistance is primarily characterized by inhibition of hepatic glucose output and glucose uptake capacity, increased blood glucose levels and circulating free fatty acids. Insulin resistance further promotes disorders of glucolipid metabolism. Glucose accumulation can stimulate the JAK/STAT, TGF-β1/SMAD, MAPKS, Jagged/Nortch pathway to promote inflammation and extracellular matrix deposition, which can lead to kidney injury. Glucose accumulation also promotes cholesterol (TC), triglyceride (TG), lipid synthesis-regulating transcription factor upregulation (SREBP), and LDL-C levels, and decreases high-density lipoprotein (HDL-C) levels, which promotes the buildup of fats in the liver and kidney tissues, resulting in kidney damage. However, the toxic effects of different lipid species on specific renal cells and their downstream signaling mechanisms have not been systematically investigated. The use of lipidomics technology to dynamically track the changes in the renal lipid profile during the course of DKD and to identify key toxic lipid molecules is beneficial in providing a theoretical basis for the development of innovative therapies targeting metabolic reprogramming in the kidney. The process by which abnormal glucolipid metabolism results in DKD is shown in Figure 2.

### 2.3 Autophagy

Autophagy is a bioregulatory process that can recognize and degrade damaged macromolecular proteins, organelles, and invading pathogens for cellular recycling to maintain cellular homeostasis (Ashrafi and Schwarz, 2013). Research has demonstrated that maintaining the homeostasis of autophagy is imperative for optimal renal function and that aberrant autophagy activity is closely associated with the development of DKD (Parmar et al., 2022; Koch et al., 2020). In typical circumstances, the degree of autophagy observed in podocytes is notably elevated relative to other renal unit cells. This elevated level of autophagy is imperative for the maintenance of podocyte physiological function (Guo et al., 2022). However, in the DKD model, the accumulation of autophagy-related protein p62 has been shown to inhibit autophagy function in podocytes, which may contribute to further disease progression (Wang et al., 2022).

Furthermore, epidermal growth factor receptor (EGFR) is widely expressed in mammalian kidney podocytes (Zeng et al., 2009). The activation of the epidermal growth factor receptor (EGFR) signaling pathway has been shown to inhibit the formation of autophagosomes, which are mediated by beclin-1 and LC3B. In addition to this effect, the activation of the EGFR signaling pathway has also been demonstrated to increase the expression of the regulatory factor Rubicon and the autophagy substrate SQSTM1. This increase in expression has been shown to attenuate cellular autophagy and contribute to the pathological progression of DKD (Li et al., 2021).

A multitude of studies have demonstrated that proximal tubular epithelial cells (PTEC) play a pivotal role in maintaining normal kidney function and tissue repair. Under normal physiological conditions, PTEC maintains the homeostatic mechanism of autophagy. In related studies, it was ascertained that p53 was activated during the development of DKD, which prompted the enhanced expression of miR-214 in renal tubules. In turn, miR-214 targets and regulates the Unc-51-like autophagy activating kinase 1(ULK1), which hinders the initiation process of autophagy, leading to impaired autophagy in PTEC and thereby affecting the repair capacity of renal tissue (Ma et al., 2020; Yildirim et al., 2021).

**FIGURE 3:**
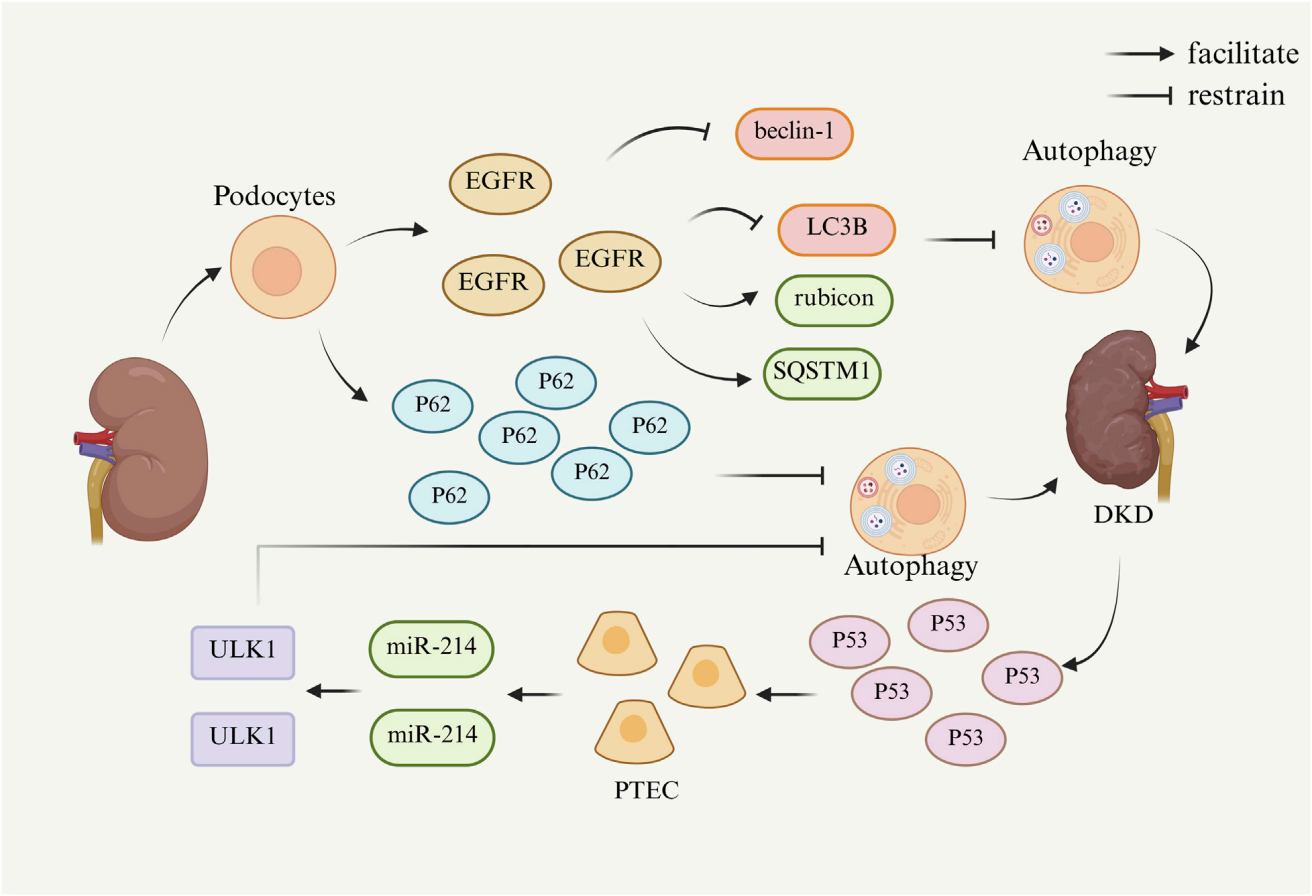
Mechanism of DKD caused by cellular autophagy. (Beclin-1, a key regulatory protein complex in autophagy; EGFR, epidermal growth factor receptor; LC3B, a specific marker for autophagosome membranes; miR-214, microRNA-214; p53, tumor suppressor gene; p62, sequestosome 1; PTEC, proximal tubular epithelial cells; Rubicon, an autophagy inhibitor; SQSTM1, sequestosome 1; ULK1, UNC-51-like autophagy-activating kinase 1).

In summary, p62 accumulates in DKD model podocytes and inhibits autophagic activity in renal podocytes. Widespread expression of the epidermal growth factor EGFR reduced the inhibition of autophagosome formation by beclin-1 and LC3B, as well as increased rubicon and SQSTM1, which ultimately inhibited cellular autophagy. p53 was activated in DKD and induced the expression of miR-214, which targeted ULK1 and hindered the initiation of autophagy, leading to impaired autophagy in PTEC. Since autophagy affects differently *in vivo* when it is at different times, it provides ideas for future studies targeting the visualization of specific cellular autophagy effects on the kidney. The mechanism by which cellular autophagy is inhibited and thus causes and promotes DKD is shown in Figure 3.

### 2.4 Inflammation response

Chronic inflammation in the kidney has been recognized as a key pathological mechanism in the development of DKD, and the inflammatory response plays an integral role in the formation and progression of renal fibrosis (RF) (Cooper and Warren, 2019).

Research has demonstrated that interleukins such as Interleukin 1 (IL-1), IL-6, and interleukin 18 (IL-18) manifest substantial expression alterations in renal tissues of DKD patients, exhibiting a close correlation with renal disease. In renal lesions, IL-1 has been shown to induce alterations in renal tissue structure by modulating the proliferation of thylakoid cells and the synthesis of matrix, as well as by increasing vascular endothelial cell permeability. These alterations, in turn, have been observed to precipitate glomerular hemodynamic abnormalities, a phenomenon attributed to fluctuations in prostaglandin levels (Royall et al., 1989; Suzuki et al., 1995). In patients with DKD, the expression level of messenger RNA (mRNA) encoding IL6 is positively correlated with the degree of dilatation of the thylakoid glands (Suzuki et al., 1995), which is a characteristic histologic feature of the disease (Fantuzzi et al., 1999). In patients diagnosed with type 2 diabetes mellitus, elevated IL18 levels in both serum and urine have been identified as an early predictive marker of renal dysfunction. The underlying mechanism may involve the MAPK signaling pathway, which is known to be activated by TGF-β (DiPetrillo and Gesek, 2004). Furthermore, elevated serum levels of tumor necrosis factor α (TNFα) have been demonstrated to be closely related to the pathogenesis of DKD, exhibiting specific pathophysiological correlations (Cortvrindt et al., 2017). The IL-17A protein has been shown to promote inflammatory responses primarily through the activation of the NF-κB signaling pathway and subsequent regulation of pro-inflammatory genes (Cortvrindt et al., 2017). In local tissues of diabetic kidney injury, elevated IL-17A can activate the expression of pro-inflammatory cytokines and chemokines (e.g., monocyte chemotactic protein-1, MCP-1) residing in renal cells, which can amplify the local inflammatory response, resulting in aggravated renal tissue injury (Ma et al., 2024). Previous studies have demonstrated that NF-κB is a downstream regulator of ALPK1 in DN renal tubular cell injury. ALPK1 activates the phosphorylation of NF-κB, and the activated NF-κB translocates from the cytoplasm to the nucleus and participates in the activation of the cellular pyroptosis pathway. In an *in vitro* study, Xinyuan Cui et al. found that downregulation of ALPK1 expression in HK-2 cells reversed the upregulation of high glucose-induced expression of cellular pyroptosis-related proteins and the increase of cellular pyroptosis (Cui et al., 2023). High glucose-induced upregulation of cellular pyroptosis-related proteins and an increase in the proportion of pyroptotic cells. Downregulated ALPK1 also decreased the renal fibrosis index α-SMA. These results suggest that activation of NF-κB is required for ALPK1-mediated cellular death to affect the symptoms of DKD.

In summary, initially, inflammatory cytokines function locally—either affecting neighboring cells (paracrine) or acting on the same cells that produce them (autocrine)—to initiate the epithelial-to-mesenchymal transition in the kidneys. Next, an increase in chemotactic cytokines and adhesion molecules draws circulating immune cells into the renal tissue, encouraging their infiltration. Ultimately, these infiltrating cells further escalate the inflammatory response by releasing additional cytokines and mediators, fueling the ongoing damage and worsening of kidney injury. In contrast, the effect of inflammatory response on DKD has received more attention from researchers. As the triggering mechanism of inflammatory response is very complex, involving the interaction of multiple cells, pathways and factors, more and more studies are working to reveal its central position and precise regulatory network in renal injury.

**FIGURE 4:**
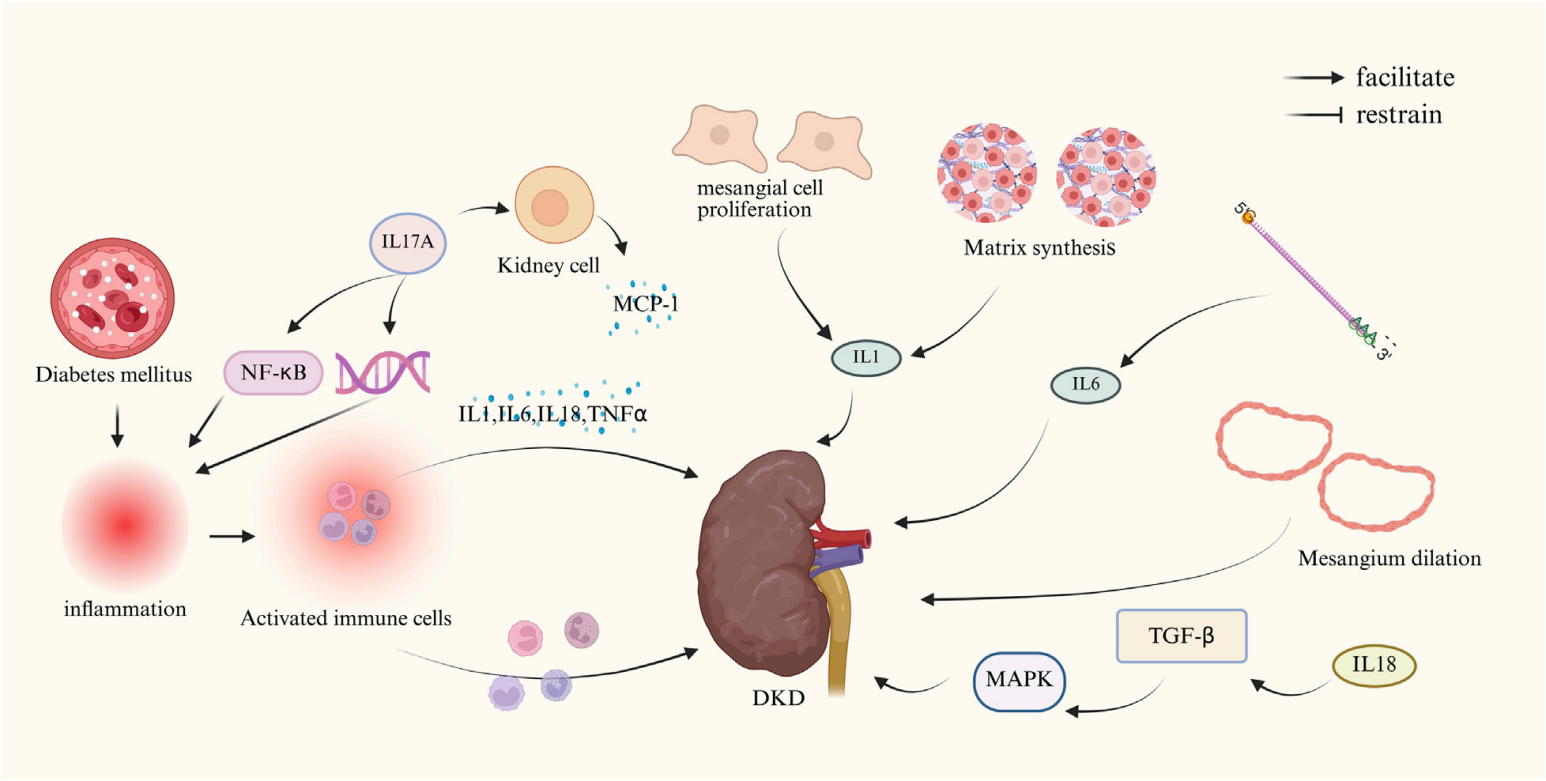
Mechanism of DKD due to inflammatory response. (IL-1, interleukin-1; IL-6, interleukin-6; IL-17A, interleukin-17A; IL-18, interleukin-18; MCP-1,monocyte chemotactic protein-1; MAPK, mitogen-activated protein kinase; NF-κB, nuclear factor-κB; TGF-β, transforming growth factor-β; TNFα, tumor necrosis factor α).

The main mechanism of action of inflammatory response to DKD is shown in Figure 4.

## 3 Therapeutic effects of bioactive metabolites in DKD

Natural active substances such as polyphenolic metabolites, peptides, polysaccharides, flavonoids, and so on, originate from nature, and have the advantages of high safety, multi-targeted action, abundant resources, and sustainable utilization, which show a great potential for the treatment of DKD (Mitra et al., 2025; Mitra et al., 2024).

**FIGURE 5:**
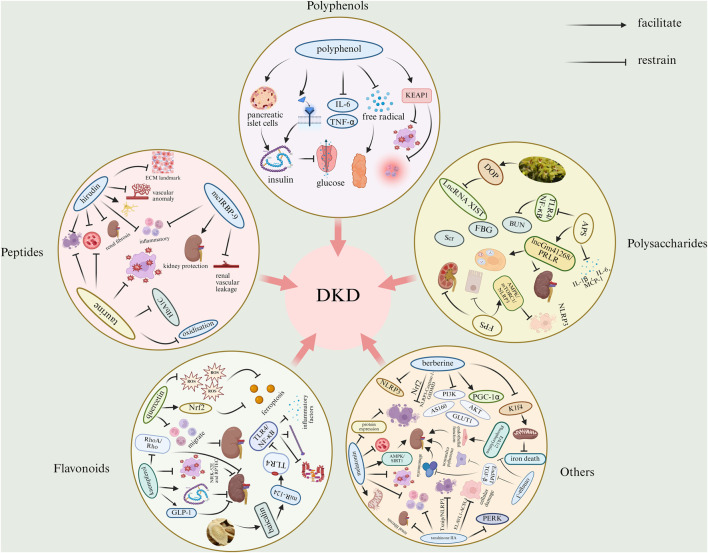
Mechanisms of bioactive substances in the treatment of DKD. (ACSL4, acyl-CoA synthetase long chain family member 4; AMPK, adenosine monophosphate-activated protein kinase; APS, astragalus polysaccharide; AS160, Akt substrate of 160 kDa; BUN, blood urea nitrogen; DOP, dendrobium polysaccharide; ECM, extracellular matrix; ELAVL1, embroyonic lethal, abnormal vision, drosophila-like 1; EndMT, endothelial-to-mesenchymal transition; ERK1/2, extracellular-regulated kinase 1/2; FBG, fasting blood glucose; FPS, fucoidan; GLP-1, glucagon-like peptide-1; GLUT1, glucose transporter 1; HbA1c, hemoglobin A1c; IL-1β, interleukin-1β; IL-6, interleukin-6; K1f4, keratin 1, type II cytoskeletal 4; LncRNA, Long non-coding RNA; MAPK, mitogen-activated protein kinase; MCP-1, monocyte chemotactic protein-1; mTORC1, mammalian target of rapamycin complex 1; miR-124, microRNA-124; NF-κB, nuclear factor-κB; NLRP3, NOD-like receptor family pyrrole-like domain 3; Nrf2, nuclear factor erythroid 2-related factor 2; KEAP1, kelch-like ECH-associated protein 1; PERK, protein kinase R-like endoplasmic reticulum kinase; PGC-1α, peroxisome proliferator-activated receptor gamma coactivator -1α; PI3K, phosphatidylinositol 3-kinase; PRLR, prolactin receptor; Rho, Rho family small GTPases; RhoA, Rho family small GTPases; ROS, reactive oxygen species; Scr, serum creatinine; SIRT1, silent information regulator 1; TGF-β, transforming growth factor-β; TLR4, toll-like receptor 4; TNF-α, tumor necrosis factor-α; XIST, X-inactivating specific transcript).

We explored the therapeutic effects of polyphenols, peptides, polysaccharides, flavonoids, and other bioactive substances in diabetic nephropathy. We found that some of the polyphenolic bioactives could enhance antioxidant enzyme activity, increase free radical scavenging capacity, and inhibit the release of inflammatory factors. Some of the polypeptides can be antifibrotic, anti-inflammatory, inhibit abnormal angiogenesis, improve apoptosis, and reduce renal vascular leakage. Some of the polysaccharides can reduce blood glucose and blood lipids, improve inflammatory response, inhibit cell death, and inhibit renal fibrosis. Some of the flavonoids can block iron death, reduce oxidative stress-related renal injury, reduce the expression of related pro-inflammatory factors, inhibit related enzyme activities, and reduce renal fibrosis. Some of the other bioactive substances are anti-fibrotic, anti-inflammatory, anti-oxidative stress, improve endothelial function and thylakoid dilatation, prevent iron death, lower blood glucose and reduce hyperglycemia-mediated pathological damage. The main mechanisms of some natural active substances for the treatment of DKD are shown in Figure 5 and Supplementary Table S1.

### 3.1 Polyphenolic metabolites

Polyphenols, characterized by their polyphenolic structures, are secondary metabolites that are widely distributed in the skin, roots, leaves, and fruits of plants (Ma et al., 2024). Numerous natural substances are common polyphenols, such as curcumin (CUR), proanthocyanidins (PA), and resveratrol (RES).

#### 3.1.1 Curcumin

Curcumin (CUR) is a bioactive polyphenolic compound found in turmeric (*Curcuma longa* L., belonging to the Zingiberaceae family), exhibiting anti-inflammatory, antioxidant, autophagy-enhancing, anti-apoptotic, and anti-fibrotic properties in DKD (6). CUR has been demonstrated to activate TFEB, which in turn boosts autophagy and lysosomal function. Curcumin in DKD lowers p-mTOR levels, which supports autophagy, decreases podocyte EMT (48), and enhances podocyte apoptosis through the Beclin1/UVRAG/Bcl2 pathway (Zhang et al., 2020). PI3K-Akt/mTOR is an important signaling pathway present in glomerular podocytes that exerts a significant influence on the regulation of podocyte autophagy. Among them, Atg5, Atg7, mTOR and LC3 were revealed to be associated with autophagy dysfunction in podocytes. It was demonstrated that curcumin-treated model rats showed increased autophagic vesicles in kidney tissues, elevated LC3 protein levels in MPC5 cells, and decreased p62, p-mTOR, p-Akt and P13K protein levels. What’s more, curcumin could ameliorate renal lesions caused by autophagic dysfunction of podocytes in high glucose levels through the PI3K/Akt/mTOR pathway. All these reports suggest that curcumin has a positive therapeutic effect on DKD. However, the high therapeutic dose of 300 mg/kg of curcumin used in the study may have potential side effects, and the low oral bioavailability of curcumin and its properties as a ubiquitous assay interfering substance make it difficult for future clinical translation. Future studies are urgently needed to overcome these translational barriers through dosage form modification or novel delivery systems.

#### 3.1.2 Proanthocyanidins

Proanthocyanidin, a polyphenolic mixture of polyphenolic compounds consisting of varying amounts of catechins or epicatechins, combined with anti-inflammatory and antioxidant properties (Al-Warhi et al., 2022). Dengpiao Xie et al. evaluated the pharmacological effects of PA on DKD and the potential research mechanisms through animal modeling tests. PA has demonstrated the ability to ease DKD by targeting several crucial pathways. The beneficial effects of PA on DKD mechanisms are linked to various factors, such as the regulation of oxidative-antioxidant balance (LPO, PCO, SOD, and GSH), and the activation of p38 MAPK and Keap1/Nrf2 signaling pathways. PA has an antioxidant function 50 times higher than that of vitamin E and 20 times higher than that of vitamin C. In the intracellular environment, it has ability to precisely activate the antioxidant defense system. Taking superoxide dismutase (SOD) as an example, PA promotes the expression of SOD-related genes, which leads to an increase in SOD synthesis and a significant increase in its activity. PA has significantly enhanced the ability of catalase (CAT) to break down hydrogen peroxide by improving the microenvironment for the reaction. In contrast, glutathione peroxidase (GSH-Px) is able to scavenge free radicals more efficiently in the presence of PA, with its increased affinity for the substrate (Al-Warhi et al., 2022). Although proanthocyanidins show strong antioxidant potential, their precise molecular targets and signaling pathways in complex organisms are not yet completely clear, and more in-depth molecular level studies are needed to elucidate them and further promote the clinical translation of proanthocyanidins.

#### 3.1.3 Resveratrol

Resveratrol, a natural polyphenolic metabolite, presents a remarkable potential in the field of DKD therapy to reduce blood glucose levels through a variety of complex and subtle pathways. Shengnan Chen et al. concluded that the most effective targets of action were PPARA, SHBG, AKR1B1, PPARG, IGF1R, MMP9, AKT1, and INSR structural domains after network pharmacology intersecting the drug targets of RES with disease targets. RSV mediates and enhances insulin sensitivity through SIRT-1 and AMPK targets to promote insulin secretion, while exerting a protective effect on pancreatic β-cells. At the same time, RES is able to modify insulin receptors and their downstream signaling molecules to make cell surface glucose transporter proteins work more efficiently, which in turn leads to better uptake and utilization of glucose by the cells, and ultimately achieves the key goal of lowering blood glucose concentration. Kubota et al. demonstrated that RSV treatment promoted AMPK activity (Koushki et al., 2024). Blocking nicotinamide adenine dinucleotide phosphate oxidase 4 (NOX4) and increasing the expression of adiponectin receptor-1 (AdipoR1) and AdipoR2 in renal tubular epithelial cells by AMPK activation and activating the AMPK-SIRT1-peroxisome proliferator-activated receptor (PPAR)-gamma coactivator (PGC)-1alpha axis and the peroxisome PPAR-alpha, reduced NOX4-derived intracellular ROS and lipotoxicity, and ameliorated OS, apoptosis and endothelial dysfunction in DN, which in turn reduced renal fibrosis and restored renal function.

RES is more prominent in its antioxidant and anti-inflammatory effects. Inside the body, it plays an active role in recognizing and neutralizing excess free radicals that attack biomolecules of various cells and cause cell damage. RES penetrates deep into cells, where it inhibits the release of inflammatory cytokines like IL-6, IL-1β and TNF-α . It should be noted that RES is also recognized as a PAINS compound, and its poor water solubility and rapid metabolism limit its pharmacological effects, making the current therapeutic evidence presenting dispersion, mechanism presenting uncertainty and adverse drug reactions, so more high-quality *in vivo* studies and clinical data are needed to verify its exact pharmacological effects and safety.

#### 3.1.4 Tea polyphenols

Tea polyphenols (TP), the main components of which are catechins, are mainly epigallocatechin (EC), epigallocatechin (EGC), epigallocatechin gallate (ECG), and epigallocatechin gallate (EGCG). In reports from animal models and human trials (Borges et al., 2016), the green tea polyphenol epigallocatechin gallate (EGCg) was shown to be effective in improving albuminuria in DKD (Hayashi et al., 2020). Daiki Hayashi et al. demonstrated that EGCg3 is “Me-induced” in the examination of DDT1-MF2 cell expression of GFP-DGKα translocation of diacylglycerol kinase α (DGKα), a marker of DGKα activation, which plays a crucial role in the improvement of DN. Meanwhile, EGCG is a potent activator of nuclear factor red factor 2-related factor 2 (NRF2), which binds to the ARE promoter and triggers the expression of downstream targets, thereby enhancing the cellular antioxidant defense system (Chen et al., 2008). Nrf2 deficiency resulted in the complete loss of all the effects of EGCG on attenuating epithelial-mesenchymal transition of renal tubular cells and protection against HG-induced oxidative damage and inflammation in MMC (Sun et al., 2017). In a molecular docking assay, it was further found that EGCG directly binds to the mouse KEAP1 protein by forming hydrogen bonds with specific residues within the KEAP1 protein, suggesting that NRF2 may play a key role in the protection of DN by EGCG through inhibition of the function of the KEAP1 protein. EGCG was able to activate renal NRF2 signaling without altering the expression of Keap1. In addition, EGCG reduced the levels of effectors of γ-GCS, NQO1 and HO-1 pathways in diabetic kidneys, the downstream targets of Nrf2, suggesting that its treatment significantly increased the cytoplasmic ratio of the nucleus to Nrf2, with the potential to enhance the protein function (Mohan et al., 2020). However, EGCG has very low oral bioavailability and is susceptible to rapid metabolism and degradation *in vivo*, making it difficult to achieve effective therapeutic concentrations in the kidney. In future studies, exact mechanistic validation can be performed to confirm its direct targets in more complex *in vivo* models and systematically assess its safety for long-term use.

### 3.2 Peptides

Peptides are bioactive substances, a general term for the 20 natural amino acids in proteins that form different peptides with different compositions and arrangements, and have a variety of biological functions. Most of the bioactives in nature come from animals. With modern research, certain peptide bioactives have shown therapeutic effects on DKD, such as hirudin, taurine and antigastric acid peptides.

#### 3.2.1 Hirudin

Hirudin is a primary active component derived from the medicinal leech (*Hirudo medicinalis* Linnaeus, belonging to the Hirudinidae family), exhibiting anticoagulant, antifibrotic, antithrombotic, and anti-inflammatory properties, and demonstrating significant protective effects on the kidneys (Liu et al., 2024). It is an essential metabolite extracted from leeches, possessing anticoagulant, antifibrotic, antithrombotic, and anti-inflammatory characteristics, offering notable kidney protection. In DKD rats, hirudin reduced caspase-3 expression while boosting the levels of RAC-α serine/threonine protein kinase, catalase, and heat shock protein HSP 90-α in kidney tissues. PENG et al. Reported that hirudin might play a direct or indirect role in regulating cellular metabolism, oxidative stress, and other mechanisms in the treatment of DKD.

Initially, in the realm of metabolic regulation, research has demonstrated that hirudin can lower blood sugar levels and glycated hemoglobin in a rat model of DKD induced by a high-fat, high-sugar diet combined with streptozotocin (STZ). Additionally, it appears to have a safeguarding effect on kidney function. Secondly, hirudin also protects the foot cells. Hirudin safeguards kidney health and prevents protein leakage by blocking the p38 MAPK signaling pathway, alleviating stress in the endoplasmic reticulum of podocytes, and mitigating PAN-induced damage to the cytoskeletal proteins within podocytes. Hirudin inhibits abnormal angiogenesis. Hirudin prevents the migration of glomerular endothelial cells triggered by high glucose levels by blocking the RhoA/p38/NF-kB signaling cascade. Additionally, it lowers the levels of angiogenesis-associated proteins such as VEGF and thrombomodulin-1, thereby helping to mitigate kidney damage in rat models.

Furthermore, hirudin has the potential to alleviate kidney damage by influencing Irf2 activity, which in turn suppresses the expression of Gsdmd, IL-1β, and IL-18. This chain of events helps to curb cellular pyroptosis and limits the secretion of proinflammatory molecules. Hirudin suppresses renal fibrosis and ECM production in STZ-induced DKD rats and HG-treated HK-2 cells through regulation of the HIF-1α/VEGF pathway. In a rat model of unilateral ureteral obstruction (UUO), hirudin significantly reduced UUO-induced ECM accumulation by modulating the expression of fibronectin, collagen III, and α-smooth muscle actin. It suppresses the inflammatory response by blocking NF-κB signaling pathways and also curbs TGF-β-driven increases in PAR1, S1PR2, and S1PR3 levels. Furthermore, it diminishes S1P/S1PR1 and S1PR2 signaling via PAR3 and alleviates TGF-β-induced processes such as epithelial-mesenchymal transition (EMT), fibrosis, and MCP-1 production in HK-2 cells. However, studies of hirudin are currently limited by small sample sizes and insufficient standardization of experimental design, resulting in an optimal therapeutic window and long-term safety of the drug that remain to be clarified. In addition, hirudin’s potent anticoagulant activity may pose a bleeding risk in the treatment of DKD, and the therapeutic window needs to be strictly defined in clinical studies. Currently, its renoprotective effect is mainly derived from animal experiments, lacking human data support, and its safety remains to be verified. In future studies, the anticoagulant effect of different doses of hirudin in treating clinical patients could be strictly monitored from a dose-effect perspective.

#### 3.2.2 Taurine

Taurine is an amino acid with pleiotropic protective properties. It is a major source of superoxide in the endothelium, leading to the separation of endothelial-type nitric oxide synthase eNOS and decreasing nitric oxide (NO) levels and expression of inducible nitric oxide synthase iNOS under hyperglycemic conditions (Ma et al., 2022). The present study showed that taurine can inhibit NOX-1 by decreasing NOX-1 activity, and that taurine can upregulate iNOS and increase NO levels to ameliorate physiological abnormalities such as inflammation and apoptosis. However, the results of the study on eNOS and iNOS expression and NO levels are controversial. These findings suggest that taurine may be an effective practical strategy for preventing renal diabetic injury. The research on the mechanism of action of taurine is mostly confined to the description of the phenomenon, and its direct molecular targets and complete signaling pathways have not been fully elucidated, which limits its precise drug development. In the future, we can deepen the research depth of the specific mechanism of taurine and conduct systematic long-term pharmacological effects and safety assessment.

#### 3.2.3 Antigastric acid peptides

Antigastric acid peptides are linear peptides containing 43 amino acids secreted by K cells in the small intestinal mucosa. Gene expression profiling revealed that mcIRBP-9 was able to improve DKD. mcIRBP-9, an antigastric acid peptide from Momordica charantia, enters the circulation of type 2 diabetic mice (db/db mice) after oral administration. Its prolonged administration significantly reduces blood glucose and HbA1c levels, thereby improving survival. The study indicated that mcIRBP-9 reduced renal vascular leakage and histopathological changes, altered pathways involved in inflammation and immune response, and improved inflammatory features of DKD in diabetic and non-diabetic mice. mcIRBP-9 also exhibited a novel anti-inflammatory activity and renoprotective capacity, which improved DKD. However, this study used an oral mode of administration, and it remains to be explored whether intact mcIRBP-9 is able to enter the bloodstream through the gastrointestinal tract and exert its biological effects *in vivo*.

### 3.3 Polysaccharides

Natural polysaccharides are macromolecules naturally occurring in plants, fungi, and algae that exhibit diverse and important biological activities such as antitumor, antioxidant, and antidiabetic effects (Zhao et al., 2020). In the search for therapeutic approaches for DKD, natural polysaccharides show promising potential for the treatment of DKD.

#### 3.3.1 Dendrobium officinale polysaccharide

*Dendrobium officinale* (*Dendrobium catenatum* Lindl., belonging to the Orchidaceae family) is a traditional Chinese botanical drug of significant value, and has been documented in authoritative pharmacological texts, such as the Chinese Pharmacopoeia. The botanical drug is rich in a variety of pharmacologically active metabolites and has demonstrated significant pharmacological effects in antitumor and immunomodulation. Dendrobium officinale polysaccharides (DOP), a principal active metabolite, have demonstrated pharmacological effects in regulating blood glucose and lipid levels in type 2 diabetic mice (Li et al., 2023a). In the pathogenesis of DKD, LncRNA XIST has been demonstrated to promote renal interstitial fibrosis through the upregulation of TGF-β1 expression. ZHANG and other scholars constructed a DKD model by using db/db mice, and gave DOP intervention through gavage (Zhang et al., 2024). The results of the study showed that DOP could significantly inhibit the expression of LncRNA XIST and TGF-β1, and thus effectively delay the DKD process. The results demonstrated that DOP could significantly inhibit the expression of LncRNA XIST and TGF-β1, thereby effectively delaying the development of renal interstitial fibrosis in DKD patients. However, DOP has a high molecular weight and poor absorption, and bioavailability is a major bottleneck in its clinical application. In addition, the complex composition of its polysaccharides may lead to unstable solubility and pharmacological effects. In future studies, modern separation techniques can be used to isolate single components for comprehensive structural characterization and analysis, to clarify which specific structural polysaccharide components play the main medicinal effects.

#### 3.3.2 Astragalus polysaccharide

Astragalus polysaccharide (APS), an active substance in Astragalus (*Astragalus membranaceus* (Fisch.) Bunge, belonging to the Fabaceae family), has been shown to have significant anti-inflammatory, antioxidant, and glucose-lowering biological functions. APS was found to improve renal inflammatory responses in DKD patients by inhibiting the TLR4/NF-κB pathway, and APS effectively reduced the levels of FBG, BUN, Scr, and renal pathological damage. In addition, APS significantly ameliorated renal injury by decreasing the expression of inflammatory cytokines IL-1β, IL-6, and MCP-1 and inhibiting TLR4/NF-κB pathway activity in DKD rats. In addition to improving the inflammatory response, APS may also play a therapeutic role by targeting the lncGm41268/PRLR pathway to promote autophagy and inhibit RF in DKD. However, as a macromolecular polysaccharide, the bioavailability of APS is extremely low, the oral absorption efficiency and its specific form *in vivo* are still unclear, and the current mechanism is not deep enough, lack of loss-of-function validation, and its purity and homogeneity also pose a challenge to the assessment of pharmacological effects. In the future, APS can be loaded into nanoparticles, lipoparticles and other novel carriers to enhance the bioavailability with efficient targeting.

#### 3.3.3 Fucoidan

Fucoidan is a type of polysaccharide found in brown seaweed, *Fucus vesiculosus* L. (belonging to the family Fucaceae), which is found in high concentrations in fucoidan plants and echinoderms. It has been demonstrated to possess a variety of biological activities, including substantial anti-inflammatory and anti-tumor properties, as well as the capacity to modulate immune function and inhibit the growth of pathogenic microorganisms (Li Y. et al., 2023). In the pathological process of DKD, FPS has been shown to have therapeutic potential. The study by CHEN and other scholars confirmed that low molecular weight FPS could improve DKD symptoms by blocking the process of EMT and renal fibrosis. Subsequent studies revealed that FPS could inhibit the NLRP3 inflammatory vesicle-mediated programmed death of podocytes by modulating the AMPK/mTORC1/NLRP3 signaling pathway, thereby effectively alleviating the process of RF in DKD (85). However, like APS, FPS is a class of polysaccharide mixtures with high structural heterogeneity, and the activities of components from different sources and molecular weights may vary significantly. In the future, structurally homogeneous fractions need to be prepared for more precise pharmacological evaluation.

### 3.4 Flavonoids

Flavonoids such as quercetin, kaempferol, myricetin, apigenin, baicalin, luteolin, hesperidin, genistein, proanthocyanidins, and kaempferol exert multi-targeted and multi-pathway effects in DKD therapy, demonstrating the significant therapeutic potential of certain flavonoids for DKD (Mitra et al., 2025; Hu et al., 2021).

#### 3.4.1 Quercetin

Quercetin is a natural flavonoid found in large quantities in vegetables and fruits, mainly in the form of glycosides, with anti-inflammatory, antiviral and antioxidant properties. Experiments have provided supporting evidence for the potential of quercetin as a new candidate for the development of iron death inhibitors for the treatment of DKD by evaluating the effects of quercetin on HG-induced renal tubular epithelial cell injury and the DKD rat model (Zhang et al., 2024). The findings suggest that high blood glucose levels promote the onset and progression of renal iron death in DKD rats, and that quercetin may help to attenuate this diabetic renal injury by decreasing ROS levels and activating Nrf2, which in turn blocks iron death. One experiment was performed by administering a single abdominal subcutaneous injection of quercetin to Sprague-Dawley rats and collecting blood and left kidneys for analysis. According to the results of this study, quercetin demonstrated substantial benefits in mitigating oxidative stress-induced renal injury, impeding the infiltration of inflammatory cells into renal tissues, and reducing the expression of intercellular adhesion molecule-1 (ICAM-1). However, the *in vivo* application of quercetin is mainly limited by its short biological half-life and low bioavailability. In future studies, quercetin’s pharmacokinetics can be improved by structural modification of quercetin and novel formulations, such as nanocrystals or phospholipid complexes, can be developed to facilitate its clinical translation.

#### 3.4.2 Kaempferol

As a flavonoid with substantial biological activity, kaempferol is present in a variety of foods, including tea, cruciferous vegetables, and various fruits. It exhibits multifaceted pharmacological effects, including anti-inflammatory, anti-oxidative stress, and anti-atherosclerotic effects. Activation of Rho kinase is associated with increased oxidative stress. Under high glucose conditions, Rho A is activated, leading to elevated intracellular oxidative stress, increased release of inflammatory cytokines (e.g., TNF-α and IL-1β) from renal tubular epithelial cells, and changes in the expression of fibronectin, CTGF, TGF-β1, collagen type IV, and E-calcineurin, which regulate calponin-mediated cell-cell adhesion, as well as increased pro-fibrotic growth factor, and expression of extracellular matrix components and extracellular matrix components. Extracellular matrix components and inflammatory cytokine expression. Quercetin inhibited oxidative stress and inflammatory factor expression by decreasing hyperglycemia-induced TNF-α and IL-1β levels and negatively regulating inflammation as well as the expression of TGF-β1 and ECM proteins, and inhibiting the activity of Rho-kinase in cells under hyperglycemic conditions. In addition, kaempferol has been demonstrated to attenuate renal injury and inhibit the onset of fibrosis by promoting the secretion of GLP-1 and insulin, as well as inhibiting RhoA/Rho kinase activity. However, the chemical properties of kaempferol have a dual role: both scavenging free radicals and exerting antioxidant effects through the donation of hydrogen atoms, but under specific conditions, the intermediate products (phenol-oxygen radicals) produced react with oxygen to produce ROS with pro-oxidant effects. Also similar to resveratrol, the chemical structure of kaempferol suggests that it may have PAINS properties. Therefore, its activity results in in vitro screening need more *in vivo* functional experiments to verify its specificity.

#### 3.4.3 Baicalin

Baicalin is a flavonoid compound derived from (*Scutellaria baicalensis* Georgi, belonging to the Lamiaceae family), and has been utilized in both the theory of traditional Chinese medicine and modern pharmacological studies. Recent studies have demonstrated that this compound exhibits a variety of biological activities, including pharmacological effects such as the inhibition of inflammatory responses, the scavenging of free radicals, and the inhibition of tissue fibrotic processes. It has now been found to possess anti-inflammatory, antioxidant and antifibrotic activities (Wang et al., 2022). An experiment investigated the effect of baicalin on RF using a streptozotocin-induced DKD mouse model and an HG-treated HK-2 human proximal tubular epithelial cell model (Zhang et al., 2020). The results indicated that baicalin could be used as a potential therapeutic agent to prevent DKD. Baicalin can increase the expression of microRNA-124 (miR-124), which specifically targets TLR4 in the proximal tubular epithelial cells of DKD mice. This effect ultimately blocked the TLR4/NF-κB signaling pathway, thereby reducing inflammatory mediator expression and decreasing the expression of type IV collagen and fibronectin in human kidney 2 cells under HG activation. However, the poor water solubility of baicalein may affect its bioavailability, which could be explored in future studies to better understand the therapeutic role of baicalein in the treatment of DKD.

### 3.5 Others

#### 3.5.1 Berberine

Huanglian (*Coptis chinensis* Franch., belonging to the Ranunculaceae family) is widely used in the treatment of diabetes, and its bioactive metabolite is mainly berberine. The positive impact of berberine in DKD could stem from its antifibrotic, anti-inflammatory, and antioxidant effects. Research indicates that berberine suppresses high glucose-triggered EMT and kidney fibrosis by blocking NLRP3 inflammasome activation (Ma et al., 2022). Berberine ameliorates DKD by restoring PGC-1α activity and energy homeostasis. Berberine also maintains mitochondrial structure and prevents iron death by inhibiting Klf4 promoter methylation and expression of iron death markers. Inhibition of the PI3K/Akt/AS160/GLUT1 pathway to control high glucose-induced abnormal proliferation and cell cycle progression in GMCs. Reducing oxidative stress by activating antioxidant Nrf2 to regulate NLRP3-Caspase-1-GSDMD signaling, thereby inhibiting pyroptosis and combating DKD inflammation-related injury. The bioavailability problem of berberine is widely recognized, and in the future its exact effect and long-term safety against DKD need to be more accurately assessed, and large-scale, high-quality preclinical and clinical studies must be conducted before clinical application.

#### 3.5.2 Tanshinone IIA

Tanshinone IIA (*Salvia miltiorrhiza* Bunge, belonging to the Lamiaceae family) is a diterpenoid quinone compound extracted from the dried roots and rhizomes of the Lamiaceae plant Salvia miltiorrhiza Bunge. Reported that the protective effect of tanshinone IIA against streptozotocin (STZ)-induced DKD may be related to the reduction of endoplasmic reticulum stress by attenuating PERK signaling activity. Tanshinone IIA protects mouse podocytes from damage and iron death caused by high glucose levels by regulating the ELAVL1-ACSL4 signaling pathway. Tanshinone IIA can inhibit focal death by modulating Txnip/NLRP3 inflammatory vesicles, regulation of TGFB1 inhibits high glucose-induced inflammation and cellular pyroptosis in renal tubular epithelial cells, may promote defense against DKD by inhibiting apoptosis caused by intracellular oxidative stress, can prevent podocyte damage in DKD by promoting autophagy and inhibiting inflammation, in part by inhibiting the PI3K/Akt/mTOR signaling pathway (Li et al., 2024), also ameliorates DKD-induced renal fibrosis by regulating miRNA-34a-5p in in vitro and *in vivo* studies. This study clearly demonstrated that tanshinone IIA exerts its protective effects by inhibiting cellular death caused by oxidative stress, but the molecular target of its direct action remains unclear. In addition, tanshinone IIA has poor water solubility, and most of its current experimental studies have been administered by injection, which is not applicable to the long-term management of DKD. Exploring its oral formulation is important for clinical translation.

#### 3.5.3 Melatonin

Melatonin is an endogenous hormone associated with the regulation of biological rhythms, and involved in regulating mitochondrial homeostasis. Studies have shown that melatonin enhances the phosphorylation of adenylate-activated protein kinase (AMPK) and promotes PTEN to induce the migration of putative kinase 1 (PINK1) and Parkin to mitochondria, which in turn triggers mitochondrial autophagy, reduces oxidative damage, and suppresses inflammatory responses. Alternatively, melatonin may mediate nephroprotective effects by upregulating the AMPK/SIRT1 axis, enhancing autophagy and mitochondrial health in patients with DKD (114). Melatonin inhibits STAT3 phosphorylation and reduces levels of aging-related proteins such as p53, p21 and p16. It also reduces the expression of proteins involved in apoptosis, such as cleaved PARP1, cleaved caspase-9 and caspase-3, ultimately playing a role in fighting cell aging and programmed cell death. It has also been shown that intervention with exogenous melatonin can lower blood glucose and attenuate hyperglycemia-mediated pathological damage. In addition, melatonin inhibits the activation of RAS and exhibits a strong antioxidant effect. Melatonin, as an endogenous hormone, has a wide range of actions. In the treatment of DKD, the focus needs to be on the safety of its long-term use and the potential effects on the systemic rhythmic system.

#### 3.5.4 Omega-3 fatty acids

Omega-3 fatty acids (ω-3), mainly α-linolenic acid, eicosapentaenoic acid (EPA), and docosahexaenoic acid (DHA), reduce blood lipids and enhance cellular function (Shahidi and Ambigaipalan, 2018). ω-3 may regulate renal hemodynamics by inhibiting renin activity, improving endothelial function, and reducing atherosclerosis (Chung et al., 2017; Giakoumis et al., 2019). Endothelial-to-mesenchymal transition (EndMT) is characterized by decreased expression of endothelial markers (e.g., CD31 and VE-calmodulin) and increased expression of mesenchymal markers. Activation of PKCβ has been determined to lead to inhibition of glomerular endothelial cell function, increased phosphorylation of Erk1/2, decreased activation of endothelial nitric oxide synthase in the glomerulus and endothelial dysfunction may lead to loss of antioxidant and inflammatory effects of nitric oxide, thereby aggravating DKD. In contrast, EPA-E inhibited the increase in Erk1/2 phosphorylation and ameliorated tethered dilatation and albuminuria by inhibiting EndMT and TGF-β-mediated renal fibrotic signaling, as well as inhibiting diabetes-induced upregulation of MCP-1 and TGF-β expression and decreasing MDA (Yasuza et al., 2021). In a supplementation clinical trial, omega-3 supplementation showed benefits in reducing albuminuria and maintaining renal function in diabetic patients with hypertriglyceridemia (Han et al., 2016). Another study confirmed that a combination of the synthetic vitamin D analog paricalcitol and ω-3 showed enhanced renoprotective effects (El-Bosh et al., 2022). However, in a major clinical trial, supplementation with vitamin D3 or ω-3 resulted in no significant difference in 5-year eGFR changes compared with placebo, suggesting that no significant efficacy of either supplementation was seen in patients’ glomerular filtration rates. Therefore, the specific efficacy of omega-3 fatty acids in targeting renal function in patients in today’s clinical settings needs to be further explored (de Boer et al., 2019). Although there are ongoing clinical trials, with long lead times and many confounding factors, their specific therapeutic mechanisms for DKD remain unclear, and more in-depth mechanistic exploration is needed in the future.

## 4 Discussion

In the pathogenesis of DKD, the article focuses on the major pathways of OS, including the polyol pathway, PKC pathway, MAPK pathway, and NF-κB pathway. There is less recent relevant literature available in the in-depth study of DKD and polyol pathways. The nuclear factor red cell 2-related factor 2 (NRF2), a key transcription regulator, has also been determined through searches to play an important role in fighting OS. It has been found that compounds from natural active ingredients such as berberine (Gao et al., 2025), TP (Sun et al., 2017), CUR (Zhu et al., 2025), and quercetin (Zhang et al., 2024) can exert antioxidant effects by modulating the Nrf2/Keap1 signaling pathway (Haung et al., 2024). For example, some polysaccharides may act by activating the AMPK signaling pathway. Astragalus polysaccharide was found to upregulate AMPK activity, increase insulin sensitivity, and reduce blood glucose levels in DKD studies. Additionally, it helps modulate enzymes involved in lipid metabolism and diminishes fat buildup within the kidneys. PPARγ is mainly involved in the differentiation of adipocytes and the regulation of lipid metabolism, and some of the naturally occurring active metabolites can regulate its activity. Core targets during cellular autophagy include AMPK/mTOR, among others (Selim et al., 2025; Ye et al., 2025). Berberine has been found to inhibit mTOR activity, promote cellular autophagy, and reduce apoptosis and fibrosis in kidney cells. The central target of the inflammatory response is mainly NF-κB (Chen et al., 2024). In DKD, factors such as hyperglycemia and OS activate NF-κB, which translocates from the cytoplasm to the nucleus. it kickstarts the production of inflammatory mediators like TNF-α, IL-1β, and MCP-1, which fuel inflammation and worsen kidney damage (Shen et al., 2021).

Although the bioactives such as polyphenols, peptides, polysaccharides, and flavonoids listed in this review have demonstrated great potential in preclinical studies for the treatment of DKD, and their multi-targeting and high safety profiles are particularly compelling, a series of formidable challenges remain in successfully translating these promising candidates into clinical applications. The primary challenge lies in their inherent pharmacokinetic properties, in particular their low oral bioavailability. Many natural compounds, such as ω-3 (de Boer et al., 2019), CUR (Sanidad et al., 2019) and RES (Jaisamut et al., 2021), have certain pharmacokinetic and bioavailability limitations. The source of ω-3, the pharmaceutical industry and medicinal chemistry have a greater impact on their bioavailability (Cholewski et al., 2018). Recent studies have found that the solid lipid nanoparticle formulation approach has significant advantages for ω-3 and improved biodistribution (Abdelhaf et al., 2025), and future research directions can be considered to improve the clinical efficacy of their drugs from the perspective of lipid carriers, as well as structural lipids (Garrastazu et al., 2018). Low gastrointestinal utilization of curcumin is a notable problem for this substance, and it has been found in studies that the oral bioavailability of curcumin can be greatly improved by drug fabrication methods such as co-administration (Garg et al., 2019), liposomes, and nanoparticles (Dei Cas and Ghidoni, 2019; Ma et al., 2019). Resveratrol has low water solubility and high intestinal membrane permeability; a small increase in solubility or the use of microemulsified drug delivery systems may significantly improve its bioavailability (Chimento et al., 2019). Second, although network pharmacology studies have suggested the complexity of their multi-targeted effects, more direct evidence at the proteomic and metabolomic level or further at the knockout level is still needed on how precisely these interacting networks regulate key pathways (e.g., Nrf2/Keap1, NF-κB, *etc.*) in DKD in complex organisms (Chen et al., 2023). Third, systematic safety evaluation is indispensable. Although studies have confirmed the therapeutic effects of natural active substances on nephrotoxicity (Zhai et al., 2024; Alhusaini et al., 2022), the “natural” properties of natural products are often misinterpreted as absolute safety, and the risk of potential organ toxicity interactions at high doses or under prolonged use has not been adequately evaluated. For example, some nephrotoxic adverse effects of RES ingestion were observed in human subjects in a previous study (Poulsen et al., 2013). Most of the current evidence comes from rodent models, whose pathophysiological processes differ from those of human DKD, and most of the existing studies focus on intervening in early lesions, and their pharmacological effects in reversing structural damage, such as glomerulosclerosis in advanced DKD, remains to be verified.

In terms of research on therapeutic approaches, although bioactive metabolites show good therapeutic potential, further in-depth studies on their pharmacological effects are needed. RES, kaempferol, and tanshinone IIA pan assay interfering compounds (PAINS) are a class of molecules that show activity in many assays by interfering with assay readings rather than by interacting specifically with the target (Baell and Nissink, 2018; Nelson et al., 2017). RES is a PAINS (Magalhães et al., 2021), and its *in vitro* assay may have limitations with false positive results. The *in vitro* experiments of kaempferol and tanshinone IIA should be further validated with RES in in vivo experiments or clinical trials.

Among the stilbene-based polyphenolic chemicals, RES has shown more pharmacological effects in recent years, and there are other chemicals in the same class of PA that need to be further investigated for their pharmacological effects in DKD. The optimal combination and dosage can be explored by studying the effects of combining natural metabolites from different sources to improve therapeutic pharmacological effects. Meanwhile, the mechanism of action of natural metabolites can be studied in depth through network analysis (Zhou et al., 2024), molecular studies (Han et al., 2024)and animal modeling experiments to clarify their targets and signaling pathways at the cellular and molecular levels, which will provide a basis for the development of more effective therapeutic drugs. In the future, research on their dosage form improvement and delivery systems, such as nanocarriers, liposomes, and other technologies, should be strengthened to enhance bioavailability and targeting. Interdisciplinary cooperation will be the key to promoting this class of substances from basic research to clinical practice.

## 5 Conclusion

This review provides insights into the pathogenesis of several important DKDs and the therapeutic roles of natural active ingredients. The pathogenesis of DKDs involves multiple complex processes, such as OS, abnormalities in glycolipid metabolism, dysregulated cellular autophagy, and inflammatory responses, which are interconnected and interact with each other and collectively drive the disease progression. Natural active ingredients, such as polyphenols, peptides, polysaccharides, flavonoids and alkaloids, show multifaceted beneficial effects in DKD treatment by acting on core targets and signaling pathways, such as MAPK, Nrf2/Keap1, NF-κB, including antioxidant, regulation of glycolipid metabolism, promotion of cellular autophagy, and inhibition of inflammatory response, which can improve renal function and alleviate renal pathologic In addition, we believe that in future research, we can further study how to improve the bioavailability of active substances, and at the same time, we can increase the technology of nanocarriers, liposomes, gene editing, *etc.*, in exploring the optimal therapeutic pharmacological effects of the combination of drugs for DKD.

## Abbreviations

DKD, Diabetic kidney disease; CKD, chronic kidney disease; DM, diabetes mellitus; ESRD, end-stage kidney disease; ROS, Reactive Oxygen Species; PKC, protein kinase C; DAG, diacylglycerol; VEGF, vascular endothelial growth factor; TGF-β, transforming growth factor-β; MAPK, mitogen-activated protein kinase; ERK, extracellular regulated protein kinases; NF-κB, nuclear factor kappa-B; IκB, Inhibitory proteins of nuclear factor kappa-B; TC, Total Cholesterol; TG, triglyceride; LDL-C, Low-Density Lipoprotein Cholesterol; HDL-C, High density lipoprotein cholesterol; TGF-β, transforming growth factor-β; SREBPs, Sterol-regulatory element binding proteins; MCP-1, monocyte chemotactic protein-1; TFEB, transcription factor EB; Irf2, transcription factor; ECM, extracellular matrix; PGC-1α, peroxisome proliferator-activated receptor γ coactivator 1alpha; ET-1, Endothelin-1; ADM, Adrenomedullin.
