# Supplementary material for: The pathogenesis of diabetic kidney disease and the therapeutic potential of bioactive substances
Source: Front Pharmacol. 2025 Nov 24;16:1669424. doi: 10.3389/fphar.2025.1669424 (PMC12682887; doi:10.3389/fphar.2025.1669424)
Supplement: Supplementary file 1 [file Table1.docx]

**Table 1 Mechanisms of action/targets and limitations of bioactive compounds in treating DKD**

| Type | Active substance | Source | Animal/Cells | Route of administration | Treatment | Mechanism of action/target | Limitation | References |
| --- | --- | --- | --- | --- | --- | --- | --- | --- |
| Polyphenols | Curcumin | turmeric | Male 5xFAD mice | Oral gavage | 50 mg/kg/day curcumin | TFEB↑, autophagy, lysosomal activity↑ | Low bioavailability: Poor solubility, low absorption rate, low stability, and rapid metabolism.  High therapeutic doses of curcumin may carry potential side effects, such as upper abdominal pain caused by the gastrointestinal system. | (6, 46-49) |
|  |  |  | Homozygous 3xTg mice | Oral gavage | 50 mg/kg/day curcumin |  |  |  |
|  |  |  | Male *Apoe* knock-out mice | Oral gavage | 200 mg/kg/day curcumin |  |  |  |
|  |  |  | Male Sprague–Dawley（SD） rats | Oral gavage | 300 mg/kg/day curcumin | p-mTOR↓, EMT↑ |  |  |
|  |  |  | Mouse podocytes (MPC5) | Medium supplement | 40μM curcumin |  |  |  |
|  |  |  | Male balb/c mice | Oral gavage | 200 mg/kg/day curcumin | Beclin1/UVRAG/Bcl2↑ |  |  |
|  |  |  | Mouse podocytes (MPC5) | Medium supplement | 20,40or 80 µM curcumin |  |  |  |
|  | Proanthocyanidin | plants | Male Kunming mice | Intraperitoneal injection | 5mg/kg/day proanthocyanidin | SOD , CAT break down hydrogen peroxide, glutathione peroxidase (GSH-Px) activity↑ | Low bioavailability: poor absorption and rapid metabolism.  The lack of conclusive research evidence limits the clinical translation of proanthocyanidins. | (19, 51-53) |
|  |  |  | Male Wistar rats | Oral gavage | 250 mg/kg/day proanthocyanidin | AGEs/RAGE axis expression, urinary albumin excretion↓ |  |  |
|  |  |  | male SD rats | Oral gavage | 250mg/ kg/day proanthocyanidin | Caspase-12 pathway↑, apoptosis↓ |  |  |
|  | Resveratrol | tiger Balm and Grapes | C57BL/KsJ-db/db mice | Oral administration | 0.005%（w/w）or0.02%(w/w)resveratrol | AMP-activated protein kinase↑, blood glucose ↓ | The evidence for treatment shows inconsistency, the mechanisms remain uncertain, and adverse drug reactions introduce additional uncertainty and conflicting factors to the hypothesis of improving renal injury in diabetic animal models.  Further research is needed for clinical translation and optimal dosing strategies.  Clinical translation and optimal dosing strategies require further investigation. | (53-57, 59) |
|  |  |  | Rat glomerular mesangial cell line（HBZY-1） | Medium supplement | 5, 10 or 20 μM resveratrol | Cell proliferation, inflammation, fibronectin↓ |  |  |
|  | Tea polyphenols | tea-leaf (plant) | Male SD rat; RAW264.7 cells | Oral gavage | 75 mg/kg,  150 mg/kg,  300 mg/kg | SIRT1 activation ↑, renal inflammation ↓, podocyte lipid accumulation ↓ | The mechanism of macrophage polarization is complex and unclear, and the role of TP in targeting macrophages for the treatment of inflammation, lipid metabolism, and cell polarization is difficult to explore and unstable. | (123) |
| Peptides | Hirudin | leech | SD rats | Subcutaneous injection | 5 U of hirudin | Inflammation, podocyte apoptosis, renal injury↓ | The study sample size was small, and randomization, blinding, or allocation concealment methods were not standardized, posing a risk of bias. Study durations varied, with insufficient data on adverse events and long-term follow-up, leaving safety to be verified.  The sample size was small, and randomization, blinding, or allocation concealment methods were not standardized, posing a risk of bias.  Heterogeneity among studies was substantial, and its sources were not fully elucidated.  Study durations varied, with insufficient data on adverse events and long-term follow-up, leaving safety to be verified. | (66-69, 124) |
|  |  |  | SD rats | Subcutaneous injection | 1 U of hirudin | HIF-1α/VEGF signaling pathway, expression of ECM markers↓ |  |  |
|  |  |  | Human immortalized renal tubular epithelial cells | Medium supplement | 10mg/mL hirudin |  |  |  |
|  | Taurine | meat and seafood | Male SD rats | Oral administration | 2%taurine solution | caspase-3, p-Akt↓, Bax, Bcl-2 expression↑ | Evidence is derived from animal studies, limiting the extrapolation of conclusions to humans.  Mechanistic research lacks sufficient depth. | (70, 71) |
|  | Antigastric acid peptide | Momordica charantia | db/db mice | Oral administration | 50or100mg/kg/day antigastric acid peptide | Blood glucose and HbA1c levels↓ Renal vascular leakage and histopathological changes↓Inflammation↓ | The transition from animal models to human applications involves uncertainties.  The mechanism of action has not yet been fully elucidated. | (72) |
| Polysaccharides | Dendrobium officinale polysaccharide | Noble Dendrobium | Male ICR mice | Oral gavage | 200, 400 or  800mg/kg/day dendrobium officinale polysaccharide | Blood sugar, blood lipids↓ | Low bioavailability: Large molecular weight hinders absorption.  Complex and heterogeneous polysaccharide composition leads to unstable solubility.  Potential mismatch between animal models and age-specific design.  Intervention period too brief (polysaccharides typically exhibit delayed onset of action). | (74, 125, 126) |
|  |  |  | db/db mice,db/m mice | Oral gavage | 400 mg/kg/day dendrobium officinale polysaccharide | LncRNA XIST, TGF-β1 expression↓ |  |  |
|  | Astragalus polysaccharide | Astmgali Radix | BALB/c mice | Oral gavage | 200 mg/kg/day astragalus polysaccharide | Activation of MAPK and NF-κB inflammatory pathways, production of inflammatory mediators and chemokines, cell necrosis and apoptosis↓ | Extremely low bioavailability.  Insufficient depth of mechanism studies: Lack of loss-of-function experiments.  Purity and homogeneity unknown. | (77-80) |
|  |  |  | Male SD rats | Oral gavage | 200, 400 or  800mg/kg/day astragalus polysaccharide | TLR4/NF-κB↓ |  |  |
|  |  |  | db/db mice,db/m mice | Oral administration | 2g/kg/day astragalus polysaccharide | lncGm41268/PRLR↑; autophagy activity↑ |  |  |
|  |  |  | rat renal proximal tubular epithelial cells | Medium supplement | 50,300 or 500 μg/mL astragalus polysaccharide |  |  |  |
|  | Fucoidan | brown seaweed, echinoderms, etc. | Male Goto-Kakizaki (GK) rats,Wistar rats | Oral gavage | 100 mg/kg/day fucoidan | TGF-β pathway, EMT, renal fibrosis↓ | Low bioavailability.  Lack of a unified paradigm for the mechanism of action.  Indirect mechanism association: Lack of direct genetic evidence. | (81, 83-85, 127) |
|  |  |  | Male SD rats | Oral gavage | 100mg/kg /day fucoidan |  |  |  |
| Flavonoids | Quercetin | vegetables and fruits | Human kidney proximal tubular epithelial cells | Medium supplement | 0,5,10,15,25 or50μM quercetin | Nrf2↑, ferroptosis↓ | Poor water solubility, poor permeability, instability in physiological media (stomach and intestines), short biological half-life, and poor bioavailability. | (87-89) |
|  |  |  | SD rats | Oral administration | 100 mg/kg/day quercetin |  |  |  |
|  |  |  | SD rats | Subcutaneous injection | 10mg/kg quercetin | ROS, inflammatory cell infiltration into the kidney, ICAM-1 expression↓ |  |  |
|  | Kaempferol | tea, cruciferous vegetables and a variety of fruits | NRK-52, RPTEC cells | Medium supplement | 5,10 or 50 μM kaempferol | RhoA/Rho kinase, NRK-52E, and RPTEC cells Oxidative stress, TNF-α, IL-1β expression↓ | Low bioavailability. Potential genotoxicity may exist. | (90-93) |
|  | Baicalin | Scutellariae Radix | RAW 264.7 macrophage cell line | Medium supplement | 60,90,120,150 or180μg/mL baicalin | Expression of pro-inflammatory cytokines and inflammatory cells, inflammation, NF-κB, and MAPK pathway proteins↓ | Poor water solubility and low bioavailability result in rapid metabolic clearance within the body, making it difficult to cross the blood-brain barrier and exert effective neuroprotective effects.  The pharmacological effects and safety for treating DKD require more precise evaluation. | (94-98) |
|  |  |  | Male BALB/c mice | Intraperitoneal injection | 25,50or100 mg/kg baicalin |  |  |  |
|  |  |  | Male C57BL/6 mice | Oral gavage | 10,20or40 mg/kg/day baicalin | T GF-β/Smad signaling pathway, renal interstitial fibrosis↓ |  |  |
| 0thers | Berberine | Coptis chinensis Franch | Human proximal tubule epithelial cells | Medium supplement | 1,10 or100 μM berberine | NLRP3 inflammasome, EMT, renal interstitial fibrosis↓ | There remains a gap between animal models and clinical translation.  Research has clarified that Danshen-tong IIA exerts its protective effects by inhibiting pyroptosis induced by oxidative stress, but its direct molecular targets remain unclear. | (99-104) |
|  |  |  | Male SD rats | Oral administration | 150 mg/kg/day berberine |  |  |  |
|  |  |  | Conditionally immortalized mouse podocytes | Medium supplement | 0.4μM berberine | PGC-1α activity and energy homeostasis↑ |  |  |
|  |  |  | Male C57BLKS/J db/db diabetic mice and their non‐diabetic littermates | Oral gavage | 200or300 mg/kg/day berberine |  |  |  |
|  |  |  | Male C57BL/6 mice | Oral gavage | 90or180 mg/kg/day berberine | PI3K/Akt/AS160/GLUT1 signaling pathway, cell proliferation↓ |  |  |
|  |  |  | Glomerular mesangial cells | Medium supplement | 7.5,15,30,60,90,120or150 μM berberine |  |  |  |
|  | Tanshinone IIA | salvia miltiorrhiza | Male SD rats | Intraperitoneal injection | 2,4or8 mg/kg/day tanshinone IIA | SOD activity↑, PERK pathway, collagen expression↓ | There remains a gap between animal models and clinical translation;  Research has clarified that Danshen-tong IIA exerts its protective effects by inhibiting pyroptosis induced by oxidative stress, but its direct molecular targets remain unclear. | (105-111) |
|  |  |  | Conditionally immortalized mouse podocyte cell line | Medium supplement | 5,10, or 20 μM tanshinone IIA | MPC5 cell damage and ferroptosis↓ |  |  |
|  |  |  | Male C57BL/6J mice ,*db*/*db* mice | Intraperitoneal injection | 10 mg/kg/day tanshinone IIA |  |  |  |
|  |  |  | Human renal tubular epithelial cell line | Medium supplement | 1,5or10μM tanshinone IIA | TGFB1, pyroptosis, inflammation↓ |  |  |
|  | Melatonin | wildlife | SD rats | Intraperitoneal injection | 3or10mg/kg/daymelatonin | AMPK/SIRT1 axis, autophagy ↑, oxidative stress, inflammation ↓ | It remains unclear whether existing senescent cells can be eliminated.  Its regulatory effect on endocrine function is transient, making it unsuitable for long-term blood glucose control.  Narrow therapeutic window: Only applicable to early-stage type 2 diabetes with residual pancreatic function; may be harmful for advanced or type 1 diabetes with insulin deficiency. | (112-115) |
|  |  |  | NRK-52E cells | Medium supplement | 25 or 50μM melatonin | Phosphorylation of STAT3, expression of senescence proteins p53, p21, and p16, expression of apoptosis proteins↓, cell death, blood glucose ↓ |  |  |
|  |  |  | Male C57BL/6J mice | Intraperitoneal injection | 2mg/kg/day melatonin |  |  |  |
|  | Omega-3 fatty acid | Deep-sea fish, some nuts and vegetable oils | For males ≥50 years of age and females ≥55 years of age, 71% were non-Hispanic White, 14% were non-Hispanic Black, and 11% were Hispanic. | oral administration | 1 g/d | Serum 25(OH)D ↓, serum PTH ↓, active omega-3 distribution ↓, serum vitamin D ↑ | Real-life trials with high success, long lead times, and numerous confounding factors reflect the therapeutic role of pharmacokinetics in targeting DKD, but the exact mechanism is unknown. | (128) |
